# Phytochemical Profile, GC-MS Profiling and In Vitro Evaluation of Some Biological Applications of the Extracts of *Origanum syriacum* L. and *Cousinia libanotica* D.C.

**DOI:** 10.3390/plants13010137

**Published:** 2024-01-03

**Authors:** Michella Dawra, Jalloul Bouajila, Marc El Beyrouthy, Patricia Taillandier, Nancy Nehme, Youssef El Rayess

**Affiliations:** 1 Laboratoire de Génie Chimique, Université de Toulouse, CNRS, INPT, UPS, F-31062 Toulouse, France; michelladawra@hotmail.com (M.D.); patricia.taillandier@ensiacet.fr (P.T.); 2 Faculty of Agricultural Engineering and Veterinary Medicine, Lebanese University, Dekwaneh P.O. Box 6573, Lebanon; nancy.nehme@ul.edu.lb; 3 Department of Agriculture and Food Engineering, School of Engineering, Holy Spirit University of Kaslik, Jounieh P.O. Box 446, Lebanon; marcelbeyrouthy@usek.edu.lb

**Keywords:** antioxidant activity, anti-acetylcholinesterase, cytotoxicity, *Cousinia libanotica*, *Origanum syriacum*, organic extracts, phytochemical profile

## Abstract

Indigenous to Lebanon, *Origanum syriacum* L. and *Cousinia libanotica* D.C. are notable plants in the Middle East, with *O. syriacum* known for its aromatic qualities and *C. libanotica* being less explored. Both plants have a significant role in traditional medicine for treating various ailments. This study aimed to evaluate the phytochemical composition and biological properties of the extracts from these plants. The extracts were obtained through cold maceration with solvents of increasing polarity. The ethyl acetate extract of *O. syriacum* exhibited the highest total polyphenol content. High-performance liquid chromatography (HPLC) identified fifteen compounds in both *C. libanotica* and *O. syriacum* extracts, whereas gas chromatography–mass spectrometry (GC-MS) analysis unveiled 179 volatile compounds. Notably, the *O. syriacum*-MeOH extract showed moderate antioxidant activity. Both plants’ methanolic extracts demonstrated significant anti-Alzheimer’s potential. The *O. syriacum*-dichloromethane and *C. libanotica*-cyclohexane extracts displayed the highest cytotoxicities against the HCT-116 cell line. For anti-proliferative activity against the Caco-2 cell line, the *O. syriacum*-methanol and *C. libanotica*-cyclohexane extracts were the most effective. This study provides valuable insights into the phytochemistry and potential therapeutic applications of extracts from these two oriental plant species.

## 1. Introduction

Lebanese plants are well known for being a rich source of therapeutic compounds with significant applications in the pharmaceutical industry, and many are widely used in the agri-food sector [1]. Currently, there is a noticeable surge in the functional food market due to heightened consumer interest. This shift has prompted studies investigating the link between food components and health [2]. Throughout history, and especially in Lebanon and the broader Middle East thanks to the abundant species across the Mediterranean basin, plant-based remedies have been a focal point in combating and treating various infections. Among these, *Origanum* species stand out as globally popular herbs, thriving in regions such as Eastern Europe, Middle Asia, and North and South America [3].

The term “*Origanum*” derived from the Greek words “oros,” signifying mountain or hill, and “ganos,” meaning ornament, translates to “ornament of the mountains.” Across antiquity, Origanum species have served as medicinal herbs. However, in contemporary times, their significance in culinary applications has surpassed their therapeutic value. In recent times, numerous species have found use as decorative plants. Linnaeus initially characterized the genus *Origanum* in 1754, and it is classified under the Lamiaceae family within the Mentheae tribe. This genus is prized for its volatile oil and exhibits remarkable morphological and chemical diversity [4]. It is crucial to administer it in proper doses, since although it imparts benefits in therapeutic amounts and short durations, excess consumption can be toxic. *Origanum* species also possess culinary and agricultural significance, encompassing ovicidal, herbicidal, and insecticidal traits, as well as being used as food spices [5,6].

A specific point of interest lies in a plant native to the Levant region: *Origanum syriacum*, commonly known as Syrian oregano, Lebanese oregano, or the hyssop of the Bible. Referred to as “za’atar” in common parlance, it is a crucial element in the za’atar mixture, which also encompasses sesame and sumac (*Rhus coriaria* L.). From a botanical standpoint, *O. syriacum* is an aromatic perennial herb that has woody roots and hairy stems and can reach heights of between 60 and 90 cm. *O. syriacum’s* aerial parts feature secretory trichomes containing essential oils, including carvacrol and thymol, both of significant medicinal value. These compounds contribute to the distinct aroma and flavor that characterize these plants [7,8]. Despite the presence of numerous phytochemical compounds in *O. syriacum* extracts, limited research has been carried out to analyze their composition. Depending on the type of extract and the nature of the solvent, different compounds have been identified as terpenoids, phenolic compounds, carotenoids, thymol, carvacrol, thymoquinone, rosmarinic acid, and ursolic acid [9,10]. The *O. syriacum* extracts showed a wide range of biological activities, including antioxidant, antimicrobial, anti-inflammatory, and anticancer effects [7].

Conversely, understanding and research regarding *Cousinia* species are scarce [5]. In traditional medicine, *Cousinia libanotica’s* aerial parts are employed for treating infections, wounds, and anemia [6]. *C. libanotica*,known as “Chawk rmédé,” falls within the Asteraceae family and is exclusively endemic to Lebanon [1,2,3,4,5,6,7,8,9,10]. It grows between altitudes of 1500 and 3000 m on Lebanese mountains [11]. In the literature, no studies can be found dealing with the chemical composition or biological activities of *C. libanotica.*

The primary objective of this research work is to explore the phytochemical profiles and biological activities of *Origanum syriacum* L. and *Cousinia libanotica* D.C. extracts obtained by cold maceration. Specifically, the study aims to identify chemical compounds, assess antioxidant potential (DPPH scavenging), and explore biological activities, including the anti-Alzheimer’s properties and the anti-proliferative effect against two cancer cell types, in extracts from both species.

## 2. Results and Discussion

### 2.1. Extraction Yields

**Figure 1:**
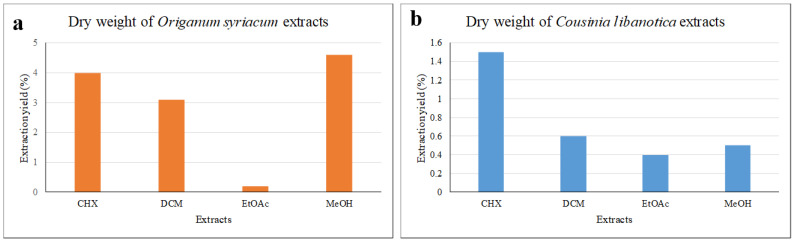
Extraction yields (%) of the eight extracts of the two plants: (**a**) *Origanum syriacum* and (**b**) *Cousinia libanotica*.

Starting from 100 g of plant samples, the resulting extraction yields for the eight extracts from *O. syriacum* and *C. libanotica* were quantified and depicted in Figure 1a,b.

*O. syriacum* demonstrated the highest yield with a MeOH extract (4.6%), followed by CHX (4%), DCM (3.6%), and EtOAc (0.2%) extracts (Figure 1a). For *C. libanotica*, the CHX extract had the highest yield (1.5%), followed by the DCM (0.6%), MeOH (0.5%), and EtOAc (0.4%) extracts (Figure 1b). Notably, this study marked the first use of these solvents in the preparation of *C. libanotica* extracts. Another study reported higher yields in MeOH extracts of different plants, including *C. ramosissima*, *C. foliosa*, *C. davisiana*, and *C. stenocephala* (10%, 8.7%, 10%, and 10%, respectively), all surpassing the values obtained in our current investigation (0.5%) [12]. The *C. stenocephala*-DCM extract exhibited a significantly higher yield (10%) than the *C. libanotica*-DCM extract (0.6%). In a separate research work, *O. syriacum* MeOH extraction produced a yield of 45.3%, which is notably higher than our investigation (4.6%) [13]. Moreover, Al-Kalaldeh et al. reported a 2.5% yield for both *O. syriacum* EtOH and water extracts [14].

### 2.2. Total Phenolic Content

No prior investigations had been conducted regarding the total phenolic content (TPC) of *C. libanotica* extracts. This study marked the first exploration into this aspect. The TPC values acquired for the eight extracts of both *C. libanotica* and *O. syriacum* are enumerated in Table 1.

In addition, the *O. syriacum* extracts, particularly the EtOAc extract, exhibited the highest TPC concentration (112.3 ± 1.5 mg GAE/g dw), followed by the methanol extract at 98.4 ± 3.1 mg GAE/g dw. The CHX and DCM extracts showed TPC amounts of 51.2 ± 1.5 and 42.4 ± 1.2 mg GAE/g dw, respectively (Table 1). After confirming the homogenous subsets, the variables were allocated into four distinct N values, revealing significant variations in the TPC values among the extracts based on the solvent used (*p* ≤ 0.05). Notably, the TPC values for polar *O. syriacum* extracts surpassed those reported by Proestos et al. [15]. Their values for *O. dictamnus* and *O. majorana* were 5.4 to 5.8 times lower with MeOH/water (60:40, *v*/*v*) and 16 to 31 times lower with EtOAc/water (60:30, *v*/*v*) compared with the current study using pure MeOH and EtOAc solvents, respectively.

The MeOH extract of *C. libanotica* exhibited the highest TPC value at 43.1 ± 8.2 mg GAE/g of dw. Notably, there was a noticeable decrease in the concentration of extractable phenolic compounds as solvent polarity decreased. The second-highest value was recorded with the EtOAc solvent, amounting to 25.4 ± 1.4 mg GAE/g of dw, followed by the DCM and CHX extracts, with quantities of 10.5 ± 2.9 and 4.2 ± 0.2 mg GAE/g of dw, respectively. Significant statistical differences in TPC values were observed among the four extracts based on the organic solvent employed. 

### 2.3. Identification and Quantification of Phenolic Compounds by High-Performance Liquid Chromatography Coupled to UV Diode Array (HPLC-PDA)

**Figure 2:**
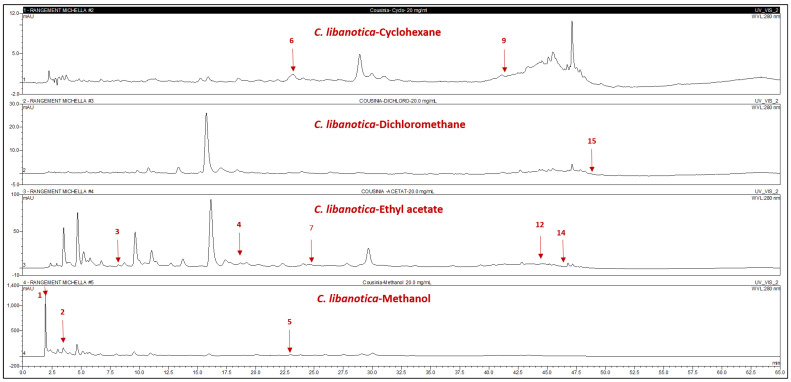
HPLC chromatograms of the cyclohexane (CHX), dichloromethane (DCM), ethyl acetate (EtOAc), and methanol (MeOH) extracts of *Cousinia libanotica.* 3-Amino-4-hydroxybenzoic acid (1); gallic acid (2); 3,4-dihydroxy-5-methoxybenzoic acid (3); L-tyrosine-7-amido-4-methylcoumarine (4); rutin (5); polydatin (6); myricetin (7); 5,3-dihydroxyflavone (9); pinostiblene (12); pinosylvin monomethyl ether (14); and 3,6,3′-trimethoxyflavone (15).

**Figure 3:**
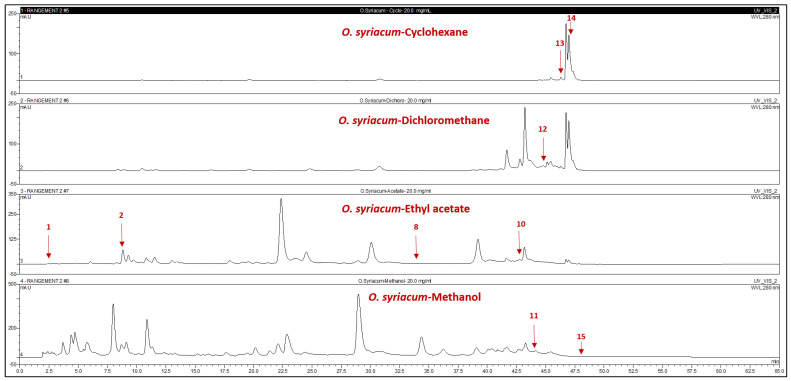
HPLC chromatograms of the cyclohexane (CHX), dichloromethane (DCM), ethyl acetate (EtOAc), and methanol (MeOH) extracts of *Origanum syriacum* L. 3-Amino-4-hydroxybenzoic acid (1); 3,4-dihydroxy-5-methoxybenzoic acid (2); 2,4-dihydroxy-3,6- dimethylbenzoic acid (8); (z)- 4-hydroxytamoxifen (10); 7-hydroxyflavone (11); pinostiblene (12); benzyl-4-hydroxybenzoate (13); pinosylvin monomethylether (14); and 3,6,3′-trimethoxyflavone (15).

Compound identification through HPLC-PDA relied on matching the HPLC retention times and DAD spectra with co-injected commercial authentic standards. The quantities of individual compounds were calculated in milligrams per gram of the respective extracts, as detailed in Figure 2 and Figure 3 and Table 2.

All identified compounds were newly discovered in *Cousinia* species and *O. syriacum* extracts. Notably, 80% of the detected compounds were polar-solvent predominant. In *C. libanotica* methanolic extract and *O. syriacum*-EtOAc extract, 3-amino-4-hydroxybenzoic acid (1) stood out at 0.5 and 0.1 mg/g, respectively. Gallic acid (2) and L-tyrosine-7-amido-4-methylcoumarin (4) were concentrated in *Cousinia* methanolic extract at 5.5 and 1.2 mg/g. Myricetin (7) appeared exclusively in *C. libanotica*-EtOAc extract at 0.45 mg/g. Compound (8) and (z)-4-hydroxytamoxifen (10) exclusively appeared in *O. syriacum* EtOAc extract at 0.1 and 5.3 mg/g. Rutin (5) and 7-hydroxyflavone (11) were, respectively, present in the *C. libanotica*-MeOH and *O. syriacum*-MeOH extracts at 0.4 and 6.01 mg/g. Both species contained pinostilbene (12) and pinosylvin monomethyl ether (14), which were most abundant in *O. syriacum*-DCM extract at 3.8 and 19.9 mg/g, respectively. Although 3,6,3’-trimethoxyflavone (15) was found in both species, the highest quantity was in *C. libanotica*-DCM extract at 0.2 mg/g. Compound (3) reached 0.4 mg/g in *O. syriacum*-EtOAc extract. Polydatin (6) and 5,3-dihydroxyflavone (9) exclusively emerged in the *C. libanotica*-CHX extract at 2.64 and 0.1 mg/g. Benzyl-4-hydroxybenzoate (13) was in both the *O. syriacum*-CHX and DCM extracts, with higher amounts in DCM (0.74 mg/g) than in CHX (0.4 mg/g). Multiple compounds were found in various extracts, depending on their solubility and polarity.

### 2.4. Gas Chromatography–Mass Spectrometry (GC-MS) Analysis of Origanum syriacum and Cousinia libanotica Extracts

In the beginning, 57 compounds were initially identified through GC-MS before derivatization. Following derivatization, this number expanded to 122 in the extracts of both species. The identification process relied on mass spectra and retention indices, as outlined in Table S1. This marks the inaugural investigation into the volatile compounds of *C. libanotica* and *O. syriacum* organic extracts. The *C. libanotica* extracts revealed the presence of 120 compounds, whereas the *O. syriacum* extracts contained 97 molecules. Intriguingly, 38 molecules were shared between the extracts of both species. In the *O. syriacum* extracts, certain volatile compounds identified in this study had previously been recognized in *O. ehrenbergii* extracts. For instance, compounds such as 4-thujanol (5—Table S1), phenol, 2,5-bis(1,1-dimethylethyl) (20—Table S1), 3,7,11,15-tetramethyl-2-hexadecen-1-ol (28—Table S1), methyl palmitate (29—Table S1), palmitic acid (31—Table S1), linoleic acid (35—Table S1), and stearic acid (36—Table S1) exhibited counterparts in *O. ehrenbergii* extracts [16]. Notably, after derivatization, most of the compounds found in *O. syriacum* extracts were already present in *O. ehrenbergii* extracts [16], with specific exceptions such as oct-1-en-3-ol (7′—Table S1), isopropyl catechol (23′—Table S1), 2,6-di-tert-butylphenol (28′—Table S1), β-thujaplicin (31′—Table S1), and others, as listed in Table S1. It is noteworthy that these compounds were identified in *O. syriacum* extracts for the first time. Furthermore, certain volatile compounds identified in *O. syriacum* extracts had previously been recognized in *O. syriacum* essential oil, including thymol, carvacrol, (+) spathulenol, caryophyllene oxide, thymoquinone, trans-sabinene hydrate, terpinene-4-ol, and trans-borneol [17,18]. The 𝜏-cadinol compound (25—Table S1) has been previously found in the essential oil of *Origanum vulgare* ssp. viride [19]. Glycerol (16′, Table S1), identified in both species, has been previously identified in the roots of *Cousinia polycephala* [20]. Several compounds were present in two or more extracts, such as β-sitosterol (48—Table S1) and epilupeol (54—Table S1). This can be attributed to their gradual release from disrupted plant cells during room temperature maceration, contingent on their polarity and solubility, influencing their quantity in each solvent.

### 2.5. Antioxidant Potency (DPPH Radical Scavenging Activity) of the Origanum syriacum and Cousinia libanotica Extracts

**Figure 4:**
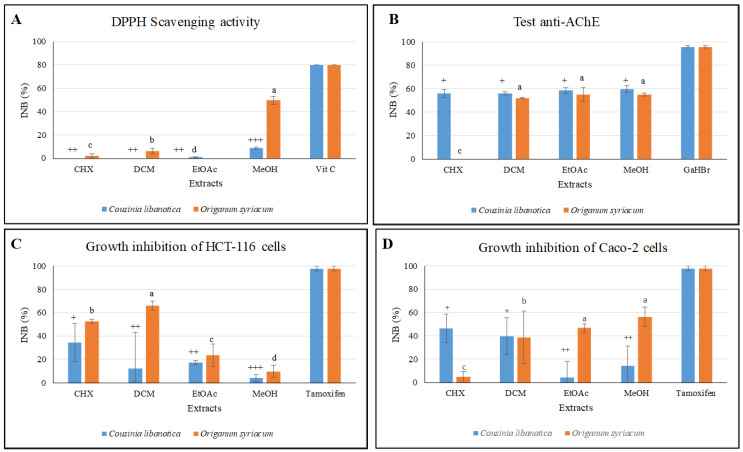
Antioxidant (**A**), anti-acetylcholinesterase “AChE” (**B**), and cytotoxic activities against two cancer cell lines: Human colorectal carcinoma “HCT-116” (**C**) and Cancer-coli “Caco-2” (**D**) of the *Origanum syriacum* and *Cousinia libanotica* extracts tested at 50 μg/mL. The inhibition percentages are compared with those of the following standards: vitamin C at 5.9 μg/mL (**A**), galanthamine dibromide (GaHBr) at 2 μg/mL (**B**), and tamoxifen at 37.1 μg/mL (**C**,**D**). The results are expressed as the inhibition percentages (% INB) and are the means of quadruplicate experiments (±SD). ^a, b, c, d, +, ++, +++^: the different superscripts represent significant differences between the values according to Tukey’s test when comparing the extracts of the same plant species (*p ≤* 0.05). The letters and the symbols are used to express the differences for the *Origanum syriacum* and *Cousinia libanotica* extracts. Cyclohexane = CHX; Dichloromethane = DCM; Ethyl acetate = EtOAc; and Methanol = MeOH.

The antioxidant potential of eight extracts from *O. syriacum* and *C. libanotica* was assessed using the DPPH assay at 50 μg/mL. As displayed in Figure 4A, MeOH extracts from *O. syriacum* (49.6%) and *C. libanotica* (8.6%) showed the highest DPPH inhibition, followed by the DCM and CHX extracts of *O. syriacum,* with percentages of 6.4% and 2.6%, respectively. Ascorbic acid, used for comparison, exhibited 80% inhibition at 5.9 μg/mL.

Conversely, the EtOAc extract of *C. libanotica* demonstrated a modest DPPH inhibition of 0.8%. Notably, the CHX and DCM extracts of *C. libanotica* and the EtOAc extract of *O. syriacum* displayed no inhibition. Statistically significant variations in the inhibition percentage values were observed among the four extracts of each tested species (*p* ≤ 0.05). Subsequent analysis involved the examination of homogeneous subsets, wherein the extracts were distributed across four distinct columns denoted as “N values” for each plant species. This approach allowed us to underscore the influence of various factors, such as solubility and polarity, on enzyme behavior. To our knowledge, previous studies have not examined the antioxidant effectiveness of organic extracts derived from *C. libanotica* and *O. syriacum.* However, Dawra et al. [16] assessed the potential of the *O. ehrenbergii*-MeOH extract in DPPH radical scavenging, revealing a 56.4% inhibition at 50 μg/mL (IC_50_ = 37.5 μg/mL), which is marginally higher than the findings in the present study.

Özer et al. [21] found *O. boissieri*-MeOH extract to have a weaker DPPH inhibitory potential, with an IC_50_ of 92 μg/mL, than the *O. syriacum*-MeOH extract in our study (49.5% at 50 μg/mL). The robust antioxidant activity of *O. syriacum*-MeOH extract is linked to its chemical composition. Utilizing advanced separation methods, such as bio-guided fractionation, could isolate individual compounds, allowing for a more precise assessment of their antioxidant activities. This approach may yield lower IC_50_ values, approaching those of the standard ascorbic acid (4.0 μg/mL). A correlation with an R^2^ value of 0.65 was established between the total polyphenol content and antioxidant activity, indicating the involvement of phenolic compounds and other molecules in the DPPH inhibition. Impressively, the *O. syriacum*-MeOH extract, which demonstrated significant antioxidant activity, contained 6.01 mg of 7-hydroxyflavone, 0.05 mg of 3,6,3′-trimethoxyflavone (Table 2), and phytol (33—Table S1), which inhibited DPPH activity by 48% at 100 μg/mL [22].

The β-sitosterol compound (48, Table S1), predominantly found in the DCM extract of *O. syriacum*, has previously demonstrated a 21.3% DPPH inhibition at 50 μg/mL [23]. Vitamin E (45, Table S1, CHX and DCM extracts of *O. syriacum*) and chlorogenic acid (107′, Table S1, MeOH extract of *C. libanotica*) displayed respective IC_50_ values of 8.3 μg/mL [24] and 3.09 μg/mL [25] against the DPPH radical. Lupeol (51, Table S1, *O. syriacum*-CHX extract) exhibited a significant dose-dependent DPPH inhibition, with an IC_50_ of 30 mg/mL, compared with quercetin (IC_50_ of 21 mg/mL) [26]. In terms of antioxidant activity, 2,6-ditert-butylphenol (*O. syriacum*-DCM extract) and syringaldehyde (*C. libanotica*-DCM extract) displayed significant and moderate effects (EC_50_: 10.3 μg/mL [27] and 1.71 mol/mol DPPH [28]). 4-Coumaric acid (*O. syriacum*-EtOAc extract) inhibited DPPH by 55.6% at 30 μg/mL [29]. Oleanolic acid (*O. syriacum*-CHX, DCM, EtOAc extracts) slightly inhibited DPPH by 8.1% at 100 μg/mL, whereas 2,4-ditert-butylphenol (*C. libanotica*-DCM extract) exhibited significant activity, inhibiting DPPH by 75% at 100 μg/mL [30]. 3,4-Dihydroxybenzaldehyde (*O. syriacum*-EtOAc extract) reduced DPPH by 92% at 40 μg/mL [31]. Compounds in the DCM extracts, including 3,7,11,15-tetramethyl-2-hexadecen-1-ol (*O. syriacum*-DCM extract) and isopropyl catechol (*C. libanotica*-DCM extract), known for having hydrogen-donor capabilities, contribute to the *O. syriacum*-DCM extract’s antioxidant activity.

### 2.6. Biological Activities of the Origanum syriacum and Cousinia libanotica Extracts

The biological properties of the extracts from *O. syriacum* and *C. libanotica* have not been previously assessed. Consequently, conducting tests on these extracts became a crucial endeavor.

#### 2.6.1. Anti-Acetylcholinesterase Activity (Anti-AChE)

The analysis was conducted using 50 μg/mL each of the *Origanum* and *Cousinia* extracts. The results were then compared with the inhibitory effects of GaHbr, which was used as the standard. Figure 4B illustrates the similar inhibition percentages for the CHX (56.1%), DCM (56.0%), EtOAc (58.3%), and MeOH (59.6%) extracts of *C. libanotica*. Likewise, the DCM (52.1%), EtOAc (54.8%), and MeOH (55.0%) extracts of *O. syriacum* showed comparable inhibitory behavior. In contrast, the CHX extract showed no inhibitory effect. Statistically, there was no significant difference between the inhibition percentage values observed among the four extracts of *C. libanotica* (*p* > 0.05). The extracts were distributed under the same N values pairwise for the non-polar extracts versus the polar extracts. This distribution suggests a similarity in the behavior of the extracts against the AChE enzyme. However, this pattern does not hold true for *O. syriacum* extracts concerning the same enzyme, where a significant difference was observed (*p* ≤ 0.05). Previous studies have explored the anti-AChE activity of six *Origanum* species: *O. boissieri*, *O. solymicum*, *O. sipyleum*, *O. saccatum*, *O. ayliniae*, and *O. hypericifolium.* In the investigation by Özer et al. [21], the most potent AChE inhibition was identified in the MeOH extract of *O. hypericifolium* (54.9%) at a concentration of 200 μg/mL. Specially, this concentration was four times higher than the one used for the MeOH extract of *O. syriacum* in our study. The latter extract, tested at 50 μg/mL, exhibited a 55.0% inhibition, indicating a fourfold more significant anti-AChE activity. The remaining five *Origanum* species demonstrated weak inhibition against the targeted enzyme. Kwon et al. [25] confirmed that chlorogenic acid (107′, Table S1), identified in the *C. libanotica*-MeOH extract in this study, exhibited AChE inhibition, with an IC_50_ of 98.17 μg/mL. Furthermore, Elufioye et al. [32] demonstrated that the presence of specific phytosterols, including campesterol (IC_50_ = 0.88 μg/mL), likely played a role in inhibiting AChE. Campesterol (110′, Table S1) was identified in both the CHX and DCM extracts of *O. syriacum* and *C. libanotica*. In a study by Heo et al. [33], naringenin was isolated as a pioneer compound and showcased a 66.0% reduction in AChE activity at 210 μg/mL. Consequently, the presence of naringenin (97′—Table S1) in the DCM and EtOAc extracts of *O. syriacum* may have contributed to AChE inhibition. Topçu et al. [34] indicated that oleanolic acid (118′, Table S1) exhibited a 50.8% inhibition of AChE at 50 μg/mL. This compound was found in the CHX, DCM, and EtOAc extracts of *O. syriacum*, suggesting its potential contribution to the anti-Alzheimer’s activity of the DCM and EtOAc extracts.

#### 2.6.2. Anti-Proliferation Activity (Cytotoxic Activity)

The inhibitory effects of *O. syriacum* and *C. libanotica* extracts prepared at concentrations of 50 μg/mL were examined on two colon cancer cell lines, HCT-116 and Caco-2. Tamoxifen was employed as a standard for comparison. In the case of HCT-116 cells, the most pronounced inhibition of cell growth was achieved using the CHX extract from *C. libanotica*, which resulted in a reduction of 34.3% (Figure 4C). This was followed by the EtOAc extract (17.4%), the DCM extract (12.1%), and finally the MeOH extract (4.2%) (*p* ≤ 0.05). Conversely, the performance of *O. syriacum* extracts on the same cell line displayed variations. The DCM extract exhibited a substantial growth suppression of 66.1% (Figure 4C), followed by the CHX extract (52.7%), the EtOAc extract (23.4%), and the MeOH extract (9.8%) (*p* ≤ 0.05). The Caco-2 cell growth inhibition was most pronounced with *C. libanotica*-CHX extract, which showed a 46.5% reduction (Figure 4D). Following this, the DCM extract displayed a 39.7% inhibition, the MeOH extract had a 14.1% inhibition, and the EtOAc extract exhibited a 4.6% inhibition. In the case of *O. syriacum* extracts, the MeOH extract demonstrated the highest inhibition of Caco-2 cell growth at 56.4%, followed by the EtOAc extract (46.4%), the DCM extract (38.6%), and the CHX extract (4.8%). Tukey’s test revealed a noteworthy difference in anti-proliferation activities among the extracts against both cancer cell lines *(p* ≤ 0.05), underscoring the statistical significance of the observed variations. There is no existing research documenting the inhibition of these two cell lines using extracts from *C. libanotica* and *O. syriacum*. Remarkably, *O. syriacum* extracts showcased promising potential as potent anti-proliferative agents against these specific colon cancer cell types. Ark et al. [35] established that retinol (70’, Table S1), which is present in the *O. syriacum* DCM extract, contributed to suppressing HCT-116 growth, resulting in an 8% reduction at 2.8 μg/mL. Jubeen et al. [36] demonstrated that the cinnamic acid derivative, 5-fluorouracil (5-FU) cinnamic acid, exhibited potent anticancer properties, inhibiting HCT-116 cell growth by 67.2% at 100 μg/mL. Syringaldehyde (41’, Table S1), which was present in the *C. libanotica*-DCM extract, was found to decrease the growth of HCT-116 and Caco-2 cells, with IC_50_ values of 56.3 and 35.9 μg/mL, respectively [37]. The D-(+)-galacturonic acid (67’, Table S1) in the *C. libanotica* MeOH extract and genistein (104’, Table S1) in the *C. libanotica* EtOAc extract have been shown to exhibit notable inhibition against HCT-116 cells, with IC_50_ values of 0.05 μg/mL [38] and 16.4 μg/mL [39], respectively. Similarly, 4-coumaric acid (54’, Table S1), which was in the *O. syriacum* EtOAc extract, reduced the number of Caco-2 cells by 43 to 75% at 164.0 μg/mL [39]. Betulinic acid (119’, Table S1), which was in the *O. syriacum* CHX and DCM extracts, has demonstrated substantial inhibition against Caco-2 cells, yielding IC_50_ values ranging between 4.4 and 16.5 μg/mL [40]. A significant reduction in cell growth has been attributed to the flavone naringenin (97’, Table S1) (present in the *O. syriacum* DCM and EtOAc extracts), which had an IC_50_ of 6.3 μg/mL [41].

### 2.7. Principal Component Analysis (PCA)

**Figure 5:**
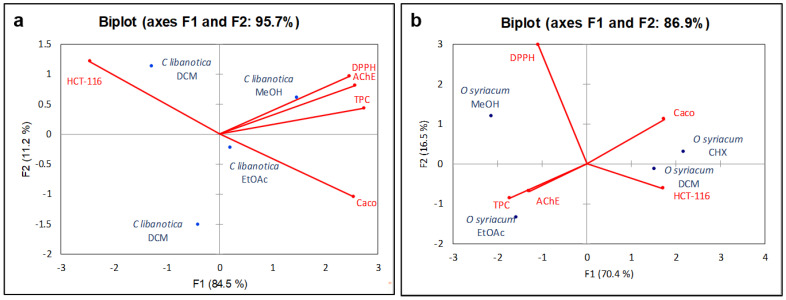
Principal component analysis “Biplot” of the total phenolic content (TPC), antioxidant properties (DPPH assay), and biological activities (anti-acetylcholinesterase AChE and cytotoxic activity against the HCT-116 and Caco-2 cell lines) for (**a**) *Cousinia libanotica* and (**b**) *Origanum syriacum* extracts. The percentages of F1 and F2 represent the most and second most variation in the data on the X-axis and Y-axis, respectively. Cyclohexane = CHX; Dichloromethane = DCM; Ethyl acetate = EtOAc; and Methanol = MeOH.

To gain a deeper insight into the relationship between the total polyphenol content (TPC) and the various biological activities assessed for both the *C. libanotica* and *O. syriacum* extracts, a principal component analysis (PCA) was employed. This analysis sought to elucidate the connections among five key components, namely the TPC, anti-AChE activity, antioxidant activity (measured by DPPH assay), and cytotoxic activity against the HCT-116 and Caco-2 cell lines, for both plant materials. As shown in Figure 5a, the first two principal components encompassed a substantial 95.7% of the data variability for *C. libanotica* extracts. The primary axis (F1) was strongly positively correlated with the total polyphenol content, antioxidant activity, anti-Alzheimer’s activity, and cytotoxicity against the Caco-2 cell line, with correlation coefficients (r) of 0.98, 0.88, 0.92, and 0.91, respectively (as shown in Table 3). For *O. syriacum*, the percentage of total variation was recorded as 86.9% and proven by the structuring accessions in Figure 5b. As listed in Table 4, F1 was strongly positively correlated with the cytotoxic activity against the two cell lines (HCT-116 (r = 0.93) and Caco-2 (r= 0.95)). F2 was only correlated with the antioxidant activity.

## 3. Materials and Methods

### 3.1. Plant Materials

In October 2018, specimens of *Origanum syriacum* L. (MNIII187c) and *Cousinia libanotica* D.C. (MNIIIb179c) were collected from Baskinta and Faraya in the Mount Lebanon governorate, situated at elevations of 1500 m and 1850 m, respectively. Dr. Marc El BEYROUTHY confirmed the identification of the plants. Herbarium samples were deposited at the School of Engineering, Holy Spirit University of Kaslik, Lebanon.

### 3.2. Extract Preparation

The harvested aerial parts of *O. syriacum* and *C. libanotica*, were air-dried in the shade at room temperature and later ground into powder. The grinding and sieving processes were performed using a coffee grinder to achieve particles of 0.8 mm. To obtain the plant extracts, the powdered plant material underwent sequential maceration with four solvents of increasing polarity (cyclohexane: CHX, dichloromethane: DCM, ethyl acetate: EtOAc, and methanol: MeOH). Each solvent extraction involved 100 g of powder mixed with 2 L of the corresponding solvent. The mixtures were agitated at 300 rpm for 2 h without applying heat. Filtrates obtained from this process were collected using Whatman filter papers (Fisher Scientific, Asin, France) and subsequently subjected to rotary evaporation under vacuum conditions at 35 °C. The resulting dried extracts were then stored at room temperature until further use.

### 3.3. Total Phenolic Content Determination

The evaluation of the total phenolic content (TPC) for each extract was carried out using the Folin–Ciocalteu (F.C) method at 765 nm, as detailed in the methodology by Dawra et al. [42]. A calibration curve was established employing the standard “gallic acid”, encompassing concentrations ranging from 0 to 115 µg/mL. The outcomes were expressed in milligrams of gallic acid equivalents (GAE) per gram of dry weight (dw).

### 3.4. Chromatographic Fingerprint Analyses using High-Performance Liquid Chromatography Coupled with Diode Array Detector (HPLC-PDA)

HPLC analysis utilized an Ultimate 3000 pump-Dionex with a Thermos Separation product DAD model detector (Thermo Fisher Scientific, Waltham, MA, USA) on an RPC18 reversed-phase column (Phenomenex, Le Pecq, France) measuring 25 cm × 4.6 mm with a 5 µm particle size. The column, maintained at 25 °C, followed the methodology from Dawra et al.’s prior work [42]. Elution occurred at 1.2 mL/min, employing MilliQ water (pH 2.6) as solvent A and acidified water/MeCN (20:80 *v*/*v*) as solvent B. A pH of 2.6 was established with ACS-grade glacial acetic acid (99.7%, Thermo Scientific Chemicals). The elution process involved a linear gradient, starting at 12% B and reaching 30% B over 35 min, progressing from 30% B to 50% B in 5 min, advancing from 50% B to 88% B in 5 min, and finally returning from 88% B to 12% B in 15 min. Samples, prepared at 20 mg/mL using the acidified water/MeCN (80:20 *v*/*v*) mixture, were filtered through a Millex-HA 0.45 µm syringe filter (Sigma Aldrich, Saint-Quentin-Fallavier, France). Injection of 20 µL of each sample followed, with detection at 280 nm. Compound identification relied on aligning the retention times and PDA spectra with co-injected commercial authentic standards and quantification used corresponding calibration curves at the maximum UV absorbance, as detailed in Table 2.

### 3.5. Gas Chromatography GC-MS Analysis

Volatile compound identification in both pre- and post-derivatization organic extracts followed Dawra et al.’s methodology [42]. The analyses used an Agilent 6890 gas chromatograph with a 5975 mass detector, and 1 µL from each extract was injected by the 7683 B autosampler. A DB-5 MS fused silica capillary column (30 m × 0.25 mm internal diameter, 0.25 µm film thickness) from Supelco (Sigma-Aldrich, Darmstadt, Germany) was employed. The column temperature started at 35 °C, increased to 85 °C at 15 °C/min, remained isothermal at 85 °C for 20 min, then rose to 300 °C at 10 °C/min and held at 300 °C for 5 min. Helium (99.99% purity) served as the carrier gas at a flow rate of 0.8 mL/min. Mass spectra were recorded at 70 eV with the ion source temperature at 310 °C and the transfer line at 320 °C, spanning from 50 to 1200 amu. The primary goal was to align the spectra with those in the NIST database, and component identification involved comparing the mass spectra with those in NIST08 (National Institute of Standards and Technology, accessed on 21 September 2021) using AMDIS software, with retention time streamlining the process. For analysis, each sample (5 mg/mL) was dissolved in its respective solvent before injection. The derivatization procedure included dissolving 5 mg of each extract in 1 mL of its corresponding solvent (excluding the MeOH extract, which was dissolved in MeCN). Subsequently, 150 µL of BSTFA and 1.5 µL of TMSC were added and, after 30 s of agitation for enhanced solubility, the mixture was kept at 40 °C for 30 min. A 10 µL portion of each derivatized solution was then injected into the GC-MS system and analyzed as previously outlined.

### 3.6. Free Radical Scavenging Activity: DPPH Test

The antioxidant scavenging capacity was assessed using the DPPH assay following Dawra et al.’s procedure [42]. In a 96-well microplate (Micro Well, Thermo Fisher Scientific, France), 20 µL of the diluted plant extract (500 µg/mL) was mixed with 180 µL of 0.2 mM methanolic DPPH solution, resulting in a final extract concentration of 50 µg/mL per well. After a 25 min incubation at room temperature, the absorbance (Asample) was measured at 515 nm. A blank, without the extract, served as the reference, and Vitamin C acted as the positive control. The DPPH inhibition percentage (% INB) was calculated as $\%INB=100\;\times \;(\frac{Ablank-Asample}{Ablank})$. All measurements were performed in quadruplicate.

### 3.7. Biological Activities

#### 3.7.1. Anti-Acetylcholinesterase Activity

The evaluation of anti-acetylcholinesterase (AChE) effectiveness followed Ellman’s protocol, as delineated by Dawra et al. [42]. In a 96-well microplate, a concoction of 50 μL of 0.1 mM sodium phosphate buffer (pH = 7.5), 125 μL of DTNB, 25 μL of thinned plant extract (500 μg/mL), and 25 μL of enzyme solution (493.2 U) underwent incubation for 15 min at 25 °C. Following the addition of 25 μL of ACTHI and an additional 25 min incubation at 25 °C, the absorbance was measured at 421 nm. A void, excluding the extract, served as the reference measurement. The enzyme activity inhibition percentage was calculated using the formula$:\;\%INB=100\;\times \;\left(\frac{Ablank-Asample}{Ablank}\right).$

#### 3.7.2. Anti-Proliferation Activity

The assessment of the cytotoxicity of the plant extracts was carried out on two distinct human colon cancer cell lines, HCT-116 and Caco-2. The anti-proliferative impact of the extracts on HCT-116 and Caco-2 cells followed the procedure outlined by Dawra et al. [11]. The cell lines were acquired from Sigma-Aldrich (Manassas, VA, USA). Each well of a 96-well microplate received 100 μL of an appropriate culture medium containing 3 × 10^^4^ cells, followed by the addition of 100 μL of the same culture medium containing the plant extract. This resulted in a final extract concentration of 50 μg/mL in each well. The employed culture media were RPMI 1640 (Sigma Aldrich, USA) for HCT-116 colon cancer cells and Dulbecco’s Modified Eagle’s Medium GlutaMAX (DMEM, Sigma Aldrich, St. Louis, MO, USA) for Caco-2 colon cancer cells. The calculation for the percentage inhibition of cell proliferation followed the formula: $\%INB=100\;\times \;\left(\frac{Ablank-Asample}{Ablank}\right).$

### 3.8. Statistical Analysis

The presented data represent the mean of four replicates with standard deviation (SD). A multiway analysis of variance was applied to the results and mean comparisons were conducted using Tukey’s multiple range test with SPSS version 20.0 (Statistical Package for the Social Sciences, Inc., Chicago, IL, United States). Significance was considered at a *p*-value < 0.05. To establish the relationship between the total phenolic content (TPC) and antioxidant or other biological activities, the linear correlation coefficient (R^2^) was calculated. For exploratory data analysis, the results underwent processing through one of the multivariate analysis techniques, namely principal components analysis (PCA). PCA was executed using XLSTAT (version 2020.1, Addinsoft, Pearson edition, Waltman, MA, USA) to enhance discrimination between the studied parameters.

## 4. Conclusions

This research project offered a comprehensive exploration into the chemical composition and a range of chemical and biological activities associated with extracts derived from *C. libanotica* and *O. syriacum* collected in Lebanon. The use of HPLC-PDA analysis helped uncover eleven previously unknown compounds in *Cousinia* species that were exclusively present in the *C. libanotica* extracts. Among these, polydatin stood out as the most abundant analyte in the CHX extract, reaching 2.6 mg/g of the extract. A similar analysis of *O. syriacum* extracts identified nine distinct molecules, with the pinosylvin monomethyl ether being the most concentrated at 19.9 mg/g of the DCM extract. These compounds comprised a range of phenolic compounds, methoxyphenols, and derivatives of *p*-hydroxybenzoic acid. Furthermore, the application of GC-MS analysis uncovered a collective total of 179 volatile compounds, of which 38 were common to both species. Specifically, 120 volatile compounds were detected in *C. libanotica* extracts, whereas 97 were observed in *O. syriacum extracts*. The *O. syriacum*-MeOH extract exhibited moderate antioxidant activity at a concentration of 50 μg/mL, yielding an activity rate of 49.6%. Additionally, both plant methanolic extracts demonstrated notable anti-acetylcholinesterase activity, achieving inhibition percentages of 59.6% for *C. libanotica* and 55% for *O. syriacum*. In terms of cytotoxic potential, the *O. syriacum*-DCM extract exhibited remarkable activity against the HCT-116 cell line, leading to a substantial growth inhibition of 66.1%. Moreover, in the case of the Caco-2 cell line, the most notable growth inhibition, reaching 56.4%, was attributed to the *O. syriacum*-MeOH extract. Considering the noteworthy biological activities observed, it is worthy of attention to undertake additional investigations to thoroughly assess the potential human health benefits and the applicability of these plant extracts in food preservation. The obtained findings motivate further exploration to identify the bioactive compounds responsible for the observed biological activities, particularly the noticeable cytotoxicity. This will involve employing silica gel fractionation followed by preparative reversed-phase chromatography. Additionally, directing research efforts towards *Cousinia* species, which have been underexplored to date, holds promise as an intriguing avenue for future studies in this field.

## Figures and Tables

**Table 1:** Expression of the total polyphenol content in milligrams of gallic acid equivalent per gram of dry weight (mg GAE/g of dw) of the eight extracts of *Origanum syriacum* and *Cousinia libanotica*.

	TPC (mg GAE/g of dw)	
Extracts	*Origanum syriacum*	*Cousinia libanotica*
Cyclohexane	51.2 ± 1.5 ^c^	4.2 ± 0.2 ^+++^
Dichloromethane	42.4 ± 1.2 ^d^	10.5 ± 2.9 ^+++^
Ethyl acetate	112.3 ± 1.5 ^a^	25.4 ± 1.9 ^++^
Methanol	98.4 ± 3.1 ^b^	43.1 ± 8.2 ^+^

^a, b, c, d, +, ++, +++^: the different superscripts in the same column represent significant differences between the TPC values according to Tukey’s test when comparing the extracts of the same species (*p* ≤ 0.05). Means values ± SD (n = 4).

**Table 2:** Quantification of fifteen compounds detected in the extracts of *Origanum syriacum* and *Cousinia libanotica* extracts by high-performance liquid chromatography coupled to photodiode array detector “HPLC-PDA” analysis.

					*Origanum syriacum* Extracts (mg of Compound/g of Extract)				*Cousinia libanotica* Extracts (mg of Compound/g of Extract)			
N°	tR (min)	λ_max_ (nm)	Compounds	Calibration Curves	CHX	DCM	EtOAc	MeOH	CHX	DCM	EtOAc	MeOH
1	2.2	281	3-Amino-4-hydroxybenzoic acid	y = 0.5995x + 0.4365			0.1 ± 0.0		0.03 ± 0.0		0.1 ± 0.0	0.5 ± 0.0
2	3.4	269	Gallic acid	y = 0.6442x − 0.4737					0.4 ±0.0		0.8 ±0.0	5.5 ±0.0
3	7.7	222	3,4-Dihydroxy-5-methoxybenzoic acid	y = 0.1682x − 0.047		0.1 ± 0.0	0.4 ± 0.1			0.1 ± 0.0	0.1 ± 0.0	
4	19.1	265	L-Tyrosine 7-amido-4-methylcoumarine	y = 0.1483x − 0.2105					0.1 ± 0.0		1.2 ± 0.0	1.2 ± 0.0
5	22.6	266	Rutin	y = 0.1029x + 0.6179								4.7 ± 0.9
6	23.3	230	Polydatin	y = 0.0445x − 0.0083					2.6 ± 0.9			
7	25.2	270	Myricetin	y = 0.1574x − 0.1168							0.4 ± 0.0	
8	35.1	340	2,4-Dihydroxy- 3,6 dimethylbenzoic acid	y = 0.1612x − 0.1498			0.1 ± 0.08					
9	42.1	286	5′,3′-Dihydroxyflavone	y = 0.1267x − 0.0317					0.1 ± 0.2			
10	43.0	330	(z) 4-Hydroxytamoxifen	y = 0.4259x − 1.3423			5.3 ± 0.1					
11	44.1	290	7-Hydroxyflavone	y = 0.1966x + 0.1052				6.0 ± 0.0				
12	44.6	240	Pinostilbene	y = 0.041x + 0.0646		11.8 ± 0.2	0.1 ± 0.0				0.3 ± 0.1	
13	46.3	300	Benzyl-4-hydroxybenzoate	y = 0.3006x + 0.0567	0.4 ± 0.0	0.7 ± 0.0						
14	47.1	257	Pinosylvin monomethyl ether	y = 0.1265x − 0.5347	17.8 ± 1.3	19.9 ± 1.4	2.0 ± 0.2			0.7 ± 0.2	0.5 ± 0.2	
15	47.9	262	3, 6,3′-Trimethoxyflavone	y = 0.1017x + 0.1091				0.1 ± 0.0	0.1 ± 0.0	0.2 ± 0.0		0.1 ± 0.0

The results are the means of duplicate experiments (±SD). Cyclohexane = CHX; Dichloromethane = DCM; Ethyl acetate = EtOAc, and Methanol = MeOH.

**Table 3:** Correlation between variables and factors for *Cousinia libanotica* plant extracts.

	F1	F2
Total polyphenol content (TPC)	0.98	0.15
Antioxidant activity (DPPH assay)	0.88	0.34
Anti-Alzheimer’s (anti-AChE)	0.92	0.29
Cytotoxic activity (HCT-116 cells)	−0.8	0.43
Cytotoxic activity (Caco-2 cells)	0.91	−0.37

**Table 4:** Correlation between variables and factors for *Origanum syriacum* plant extracts.

	F1	F2
Total polyphenol content (TPC)	−0.93	−0.22
Antioxidant activity (DPPH)	−0.59	0.79
Aanti-acetylcholinesterase (anti-AChE)	−0.70	−0.18
Cytotoxic activity (HCT-116)	0.93	−0.16
Cytotoxic activity (Caco2)	0.95	0.29

## Data Availability

Data are contained within the article and supplementary materials.

## Supplementary Materials

The following supporting information can be downloaded at: https://www.mdpi.com/article/10.3390/plants13010137/s1, Table S1: Identification of the volatile compounds of Origanum syriacum and Cousinia libanotica extracts by Gas Chromatography-Mass spectrometry“GC-MS” before and after derivatization.
