# Supplementary material for: Phytochemical Profile, GC-MS Profiling and In Vitro Evaluation of Some Biological Applications of the Extracts of Origanum syriacum L. and Cousinia libanotica D.C
Source: Plants (Basel). 2024 Jan 3;13(1):137. doi: 10.3390/plants13010137 (PMC10780604; doi:10.3390/plants13010137)
Supplement: Supplementary file 1 [file plants-13-00137-s001.zip › plants-2762888 Supplemantary Materials.pdf]

(Article)

Phytochemical profile, GC-MS profiling and *In vitro* evaluation of some biological applications of the extracts of *Origanum syriacum* L. and *Cousinia libanotica* D.C.

Michella Dawra [1,3], Jalloul Bouajila [1,*], Marc El Beyrouthy [2], Patricia Taillandier [1], Nancy Nehme [3] and Youssef El Rayess [2,*]

[1] Laboratoire de Génie Chimique, Université de Toulouse, CNRS, INPT, UPS, Toulouse, France
[2] Department of Agriculture and Food Engineering, School of Engineering, Holy Spirit University of Kaslik, B.P. 446, Jounieh, Lebanon
[3] Faculty of Agricultural Engineering and Veterinary Medicine, Lebanese University, Dekwaneh, Lebanon
* Correspondence: jalloul.bouajila@univ-tlse3.fr. (J.B); youssefrayess@usek.edu.lb (Y.E.R) ; Tel.: +961-766-43765 (Y.E.R.)

Supplementary materials

Table S1. Identification of the volatile compounds of *Origanum syriacum* and *Cousinia libanotica* extracts by Gas Chromatography-Mass spectrometry"GC-MS" before and after derivatization.

			Origanum syriacum extracts				*Cousinia libanotica* extracts			
N°	RI	Compounds	CHX	DCM	EtOAc	MeOH	CHX	DCM	EtOAc	MeOH
1	-	heptane, 2,2,4,6,6-pentamethyl-	+							
2	-	isoterpinolene	+							
3	1009	O-cimene	+							
4	1014	eucalyptol	+							
5	1063	4-thujanol	+							
6	1105	trans-sabinene hydrate	+							
7	1114	α-monoacetin		+			+			
8	1164	trans-borneol	+							
9	1194	terpinen-4-ol	+							
10	1218	cuminal					+	+	++	
11	1223	thymoquinone	+	+						
12	1224	duroquinone						+	++	
13	1249	bicyclohexane	+				+			
14	1254	benzene, 2-methoxy-1,3,5-trimethyl-						+		
15	1256	thymol	++	++	+					
16	1259	carvacrol	++	++	+					
17	1408	2,3,3-trimethylpentane							+	

18	1417	*trans*-caryophyllene	+	+						
19	1434	cuminyl acetate							+	
20	1524	phenol, 2,5-bis(1,1-di-methylethyl)-	+		++					
21	1574	*p*-cymene-2,5-diol		+	+	+				
22	1585	(+) spathulenol	+							
23	1588	caryophyllene oxide	+++	+						
24	1613	humulene oxide II	+							
25	1646	Cadinol T	+							
26	1670	14-hydroxycaryo-phyllene	+	+						
27	1785	loliolide		+						
28	1883	3,7,11,15-tetramethyl-2-hexadecen-1-ol		++	++					
29	1930	methyl palmitate								+++
30	1937	7,9-di-tert-butyl-1-ox-aspiro(4,5)deca-6,9-diene-2,8-dione	+	+++	+		+	++	+	
31	1978	palmitic acid						++	+++	
32	2005	Ethyl palmitate		+++	+				++	
33	2115	phytol	+	+	+	+++				
34	2144	pregna-4,16-diene-3,20-dione	+							
35	2154	linoleic acid	+	+	+			+++	+++	+
36	2156	stearic acid	+	+++	+				+	
37	2160	oleic acid		+				+	++	
38	2442	phenol, 2,2'-meth-ylenebis[6-(1,1-di-methylethyl)-4-methyl-	++	+++	+++	++		+++	++	+++
39	2527	2-monopalmitin		+		++				
40	2713	heptacosane	+	+			+++	++	+	

41	2725	β-monostearin				++				
42	2727	α-monostearin		+						
43	2921	octacosane	++	++						
44	2950	hexatriacontane	+++	+++	+					
45	2963	vitamin E	+	+	+			+	+	
46	3014	tetratetracontane	+++	+++	+					
47	3063	γ-sitosterol					+	+	++	
48	3070	β-sitosterol	+	+++	++		+	++	++	
49	-	β-amyrin					+++	+	+	
50	-	stigmastan-6,22-dien, 3,5-dedihydro-							+	
51	-	lupeol	+				+++	+		
52	-	stigmasta-3,5-diene							++	
53	-	β-amyrin acetate					+		+	
54	-	epilupeol					+	++	+	
55	-	3-epilupeol							+++	
56	-	lupeol acetate					+			
57	-	epilupeol acetate					+	+	+	
			**After derivatization**							
			Origanum syriacum				***Cousinia libanotica***			
N°	tR (min)	Compound	CHX	DCM	EtOAc	MeCN	CHX	DCM	EtOAc	MeCN
1′	8.1	propylene glycol						+	+	
2′	8.2	cyclohexanol	+							
3′	9.1	2,3-butane diol		+				+		
4′	9.7	carbamic acid					+			
5′	10.0	hexanoic acid	+				+	++		
6′	10.3	glycolic acid						+		
7′	10.46	oct-1-en-3-ol	++	+				+		
8′	10.48	1-butoxy-2-propanol					+			
9′	11.2	octan-3-ol	+	+						

10′	12.4	levulinic acid						+	+	
11′	13.1	benzyl alcohol						+		
12′	15.2	acetin, bis-1,3-tri-methylether			+				+	
13′	19.3	benzoic acid						+	+	
14′	21.7	octanoic acid						+		
15′	24.2	4-isopropylphenol					+			
16′	25.1	glycerol		+	+	+		+	+	+
17′	31.2	diacetin, 2-trimethylether			+				+	
18′	31.8	2-butenedioic acid, (E),						+		+++
19′	32.1	nonanoic acid	+				+	+	+	
20′	32.2	cuminol	+	+			+	++	+	
21′	35.1	decanoic acid						+	+	
22′	35.2	4'-hydroxyacetophenone						++	+++	
23′	36.0	isopropyl catechol		+						
24′	36.1	2-tert-Butyl-4-methylphenol						+		
25′	36.2	malic acid						+++		+++
26′	36.3	propyl gallate					+++	++	++	
27′	36.6	vanillin						+	+	
28′	36.8	2,6-di-tert-butylphenol		+						
29′	36.83	2,4-di-tert-butylphenol						+		
30′	36.9	myrtenoic acid					+	+	+	
31′	37.0	β-thujaplicin	+							
32′	37.3	tert-Butylhydroquinone		+						
33′	37.4	allyl carbamate							+	
34′	37.6	4-hydroxy-α-methylene-benzenemethanol						+		
35′	37.9	3,4-dihydroxybenzalde-hyde			+					
36′	38.1	4-hydroxybenzoic acid			+			+	+++	+++

37′	38.2	5-(7a-isopropenyl-4,5-dimethyl-octahydroinden-4-yl)-3-methyl-pent-2-en-1-ol	+	+					+	
38′	38.3	4-(acetyloxy)-3-methoxybenzoic acid							+	
39′	38.4	dodecanoic acid					+	+		
40′	38.9	L-(+)-lactic acid	+							
41′	39.1	syringaldehyde						+		
42′	39.2	homovanillyl alcohol						+		
43′	39.3	9-hydroxynonanoic acid						+		
44′	39.7	L-(-)-arabitol								+++
45′	39.9	vanillic acid		+				++	+	
46′	40.2	azealic acid					+	++		
47′	40.7	myristic acid	+++	+			+++	+++	+++	
48′	41.1	3-phenyllactic acid							+	
49′	41.2	quininic acid				+				
50′	41.3	glyoxylic acid monohydrate							+	
51′	41.4	syringic acid			+			+	+	
52′	41.47	D-(-)-fructopyranose								+
53′	41.6	β-D-(+)-talopyranose								+
54′	41.7	4-coumaric acid			+					
55′	41.78	pentadecanoic acid	+	+			+	++	++	+
56′	42.0	mannitol								+++
57′	42.2	myo-inositol								+++
58′	42.4	talose								+++
59′	42.5	palmitelaidic acid	+	+			+	+	+	
60′	43.3	tuberonic acid		+	+					
61′	43.6	heptadecanoic acid						+		

62′	43.68	methyl α-D-gluco-furanoside								+
63′	43.7	caffeic acid			+					
64′	43.8	methyl galactoside (1S,2R,3S,4S,5R)-								+
65′	44.3	α-linolenic acid	+	+	+		+	+		
66′	45.3	moracin M		+						
67′	45.9	D-(+)-galacturonic acid								+
68′	46.0	thymol-B-D-glucopyra-noside								+
69′	46.13	andrographolide		+						
70′	46.16	retinol		+						
71′	46.18	arachidic acid		+			++	++	+	
72′	46.3	prostaglandin E2	+							
73′	46.37	triptophenolide		+						
74′	46.93	heneicosanoic acid					+	+		
75′	47.13	2-palmitoylglycerol			+		+		++	
76′	47.17	1,3-dipalmitin							+	
77′	47.3	1-monopalmitin	+	+	+++		++	++	++	+
78′	47.5	β-arabinopyranose								+
79′	47.6	aucubin								+
80′	47.67	behenic acid	+++	+			+	+++	+	
81′	47.8	(10E,15Z)-9,12,13-trihy-droxyoctadeca-10,15-dienoic acid		+	+			+		
82′	47.9	D-(+)-turanose								+
83′	48.1	sucrose								+++
84′	48.2	maltose (isomer 2)								++
85′	48.29	lactulose								+
86′	48.3	tricosanoic acid						+		
87′	48.4	tetracosanol					+	+	+	

88′	48.5	2-monostearin			+					
89′	48.58	1-monooleoylglycerol					+			+
90′	48.6	1-monolinolein						++		
91′	48.63	1,5-anhydroglucitol								+
92′	48.69	D-arabinose								++
93′	48.7	glycerol monostearate	++	+++	+++		++	++	++	
94′	48.79	monostearin								+++
95′	48.92	lactose								+++
96′	48.93	15-tetracosenoic acid, (Z)-						+		
97′	49.0	(±)-naringenin		+	+++					
98′	49.1	lignoceric acid	+				+	++	+	
99′	49.76	pentacosanoic acid						+		
100′	49.8	1-hexacosanol	++				+			
101′	50.4	hexacosanoic acid					+	++		
102′	50.6	nonacosan-10-ol	+							
103′	50.7	5,7,4'-trihydroxy-8-c-prenyldihydro-flavonol			+					
104′	50.8	genistein							+	
105′	50.9	5-hydroxy-7-methoxy-2-methylisoflavone			+++					
106′	51.1	1-octacosanol					+			
107′	51.3	chlorogenic acid								+
108′	51.36	cholesterol					+			
109′	51.8	octacosanoic acid					+	++		
110′	52.2	campesterol	+	+			+	+		
111′	52.4	homoeriodictyol chalcone		+						
112′	52.5	stigmasterol	+++	+			+	+		
113′	52.7	1-triacontanol					+	+		
114′	53.6	α – amyrin					+++	+		

115′	54.8	erythrodiol	+					
116′	55.2	uvaol	+	+				
117′	55.4	betulinic alcohol	+					
118′	55.7	oleanolic acid	+++	++	+			
119′	55.9	betulinic acid	+++	+				
120′	56.0	lup-20(29)-en-3-ol, acetate, (3β)-					+	+
121′	56.3	ursolic acid	+	+	+			
122′	59.9	pomolic acid		+				

Note: +++: high amount; ++: moderate amount; + : low amount; RI is the retention index determined in regards to a series of n-alkanes (C7–C35) on the apolar DB-5 MS. The RI values are identified as following: $RI = 100x\ n + 100x\ ((Apex\ Rt_{(lower\ alkane)} - Rt_{(lower\ alkane)}/Rt_{(follower\ alkane)} - Rt_{(lower\ alkane)}))$ where n is the number of carbon of lower alkane. The tR is the retention time in minute. Cyclohexane = CHX, Dichloromethane = DCM, Ethyl acetate = EtOAc, acetonitrile = MeCN
